# Interactive Effects of Genes and Environment on Vegetative and Reproductive Tradeoffs of Leaf Size Mutants

**DOI:** 10.1002/pei3.70195

**Published:** 2026-08-04

**Authors:** Tina Dong, April Bisner, Leila Fletcher, Genevieve S. Depke, Josh Fentress, Christian Hamner, Weini Lin, Nicole J. Taylor, Malik White‐Hayes, Matthew T. Rutter, Allan E. Strand, Courtney J. Murren

**Affiliations:** ^1^ Department of Biology College of Charleston Charleston South Carolina USA; ^2^ Biology Department Southern Oregon University Ashland Oregon USA; ^3^ Department of Biology Barnard College New York USA; ^4^ Department of Biology Hampden‐Sydney College Hampden‐Sydney Virginia USA; ^5^ College of Biological Sciences University of California‐Davis Davis California USA; ^6^ Department of Biology Benedict College Columbia South Carolina USA

**Keywords:** *Arabidopsis thaliana*, light response curve, mutant, phenotypic plasticity, reproductive output, rosette leaves

## Abstract

Insights into phenotypic plasticity in leaf traits provide context for how plants maintain adequate resource acquisition across changing environments. Whether insertion mutations that influence early seedling rosettes translate into differences in reproductive success and phenotypic plasticity across nutrient and temperature environments is less known. We selected *Arabidopsis thaliana* mutant lines associated with a single insertion mutation in a common genetic background from a screen that categorized rosettes into two sets based on qualitative seedling size (increase or decrease) in comparison to wildtype and natural accessions. Our experiment under nutrient and temperature treatments showed the decrease set of mutant lines maintained smaller rosettes at bolting across environments, whereas the same was not found for the increase set, which revealed greater within‐set variation. Nutrients increased fruit production, particularly at 20°C, whereas the warmer temperature (24°C) increased fruit abortion and tended to reduce fruit production, with stronger effects in the increase set. For the mutant lines studied for physiology, we found CO_2_ assimilation patterns were broadly similar across temperatures despite rosette size differences, suggesting that this aspect of physiology does not explain rosette differences. Treatment responses were not parallel across sets of lines, indicating substantial among‐line variation. The variation in phenotypic traits across abiotic environments in single gene‐insertion mutants offers new quantitative insights into tradeoffs between rosette and reproduction. With data on genetic mechanisms behind plasticity, our results highlight how individual mutations can generate context dependent variation in vegetative and reproductive traits and trait relationships, useful for informing other plants' responses to climate change.

## 1 Introduction

Variation in resource acquisition for plants has long been of interest to evolutionary ecologists, and leaves are an essential component of carbon acquisition sources. Leaf size, morphology, and number contribute to a plant's capacity to acquire resources through photosynthesis and physiological function (Loveys et al. 2002; Petrík et al. 2023). Annual plants in particular, due to their short life cycle, require fast systems to obtain resources for reproduction. For winter annuals, a rosette of leaves that emerges early in the growing season is one such approach. Using a variety of methods, a growing body of literature demonstrates genetic differentiation among natural accessions on rosette leaf size and leaf number (Pérez‐Pérez et al. 2002; Alemán‐Báez et al. 2022; Fletcher et al. 2022) and the influence of essential components of the abiotic environment for plant growth, including temperature and soil nutrient availability, on these traits (Meinzer et al. 1985; Meekins and McCarthy 2000). Although much work has been completed connecting early life traits to reproductive fitness in naturally varying populations of annual plants, few studies have quantified through reverse genetic screens how single genes that impact qualitative seedling sizes influence rosette leaf size at reproductive maturity and ultimately reproductive output. Such targeted individual gene‐focused studies require investigation from ecologically informed abiotic environments as context may shift trait expression among genotypes that differ by one insertion mutation.

With limited resources, plants allocate carbon resources between growth, survival, and reproduction (Gnan et al. 2017). Flowering time and vegetative size can show tradeoffs (Bolmgren and Cowan 2008) or coordinated enhancement leading to greater reproductive output (Samis et al. 2012). Greater rosette size contributes to the capacity to synthesize more carbon resources, making these resources available for reproductive output and thus enhancing fitness (Mitchell‐Olds 1996; Baker et al. 2005; Neytcheva and Aarssen 2008). Alternatively, with earlier flowering, plants may be able to more quickly take advantage of limited resources at the expense of vegetative size and number of offspring to ensure some seed production before seasonal conditions change (e.g., Gnan et al. 2017). Phenotypic changes in responses to environmental cues allow for plants to react dynamically and enhance the expression of traits for fitness and survival to environmental variation (Callahan and Pigliucci 2002; El‐Soda et al. 2014; Acasuso‐Rivero et al. 2019). Approaches to these questions have been focused on ecological variation (Palacio‐Lopez et al. 2020), on molecular genetic sequence variation, or among population differentiation with a particular focus on genes involved in onset of flowering, as important component of these tradeoffs (e.g., Mitchell‐Olds 1996; Koornneef et al. 1998; Dijken et al. 2004; Méndez‐Vigo et al. 2022). This variation motivates examining how individual gene mutations identified from studying relative seedling size modify rosette size‐reproduction relationships across nutrient and temperature treatments.

The annual plant *Arabidopsis thaliana* is one such system where both the genetic tools and the ecological context of environmental variation exist (Weigel 2011), for a plant with a life cycle amenable to manipulative experiments through reproductive maturity. Large collections of available single mutation lines in the same genetic background provide a valuable resource for systematic examinations of individual genes on phenotypes of interest (e.g., O'Malley and Ecker 2010). These systematic examinations of gene function provide opportunities for identifying targets for studies in species with larger genomes, longer life cycles, those important for agriculture, particularly ones closely related (Zeng et al. 2025; McDormand and Qaderi 2025), or ecological settings (Koch 2018).

Databases with collections of gene‐indexed *A. thaliana* mutants have allowed for efficiency in reverse genetics to study individual gene function in a common genetic background (O'Malley and Ecker 2010; Rutter et al. 2019). One such example of this approach is in a study focused on flowering time (Chong and Stinchcombe 2019). Another is the characterization of the relative leaf size and leaf development characteristics at the seedling stage, resulting in a database of qualitative rosette leaf forms (i.e., PhenoLeaf, Wilson‐Sánchez et al. 2014). The PhenoLeaf effort was a reverse genetic screen of Arabidopsis mutants in a common agar plate and growth chamber environment. Seedlings from single gene effects were screened for seedling traits including relative rosette size, leaf color, and leaf display with a set of qualitative ontologies to aid in the continued study of gene function in the plant biology community (Wilson‐Sánchez et al. 2014). Cataloging obvious observable seedling phenotypes into qualitative groupings based on the relative difference from the control Col‐0 (the wildtype parental line for the set of mutants) in a common growth environment (e.g., Bouché and Bouchez 2001; Rutter et al. 2019) is a first step.

However, phenotypes of mutants may be masked or only expressed under certain environmental conditions (e.g., Murren et al. 2020), and trait relationships and tradeoffs also vary plastically across environments (Callahan and Pigliucci 2002). Two essential plant environments, soil nutrient availability and temperature both influence size of rosette leaf traits (Bonser et al. 2010; Gao et al. 2020; Zandalinas and Mittler 2022). Plasticity of rosette and floral traits to respond to rapid changes in these environmental conditions are crucial to maintain natural plant populations as well as crop survival and yield. Temperature influences many aspects of plant growth and function from leaf size and development, to physiological processes such as photosynthesis, to flowering time (Parent et al. 2010; Vile et al. 2011; Waraich et al. 2012; Fletcher et al. 2022). Even temperature changes of only a handful of degrees as predicted from climate models (IPCC 2021), or a few degrees in latitude during flowering (e.g., Dittmar et al. 2014) can lead to timing and morphological trait responses. These responses can vary through developmental stages and across genotypes (Morón‐García et al. 2022; Vasseur et al. 2011; Hüner et al. 2013; Prinzenberg et al. 2020) and can be plastic and locally adapted (Debieu et al. 2013). Similar to temperature, modest variation in availability of nutrients, particularly nitrogen, phosphorus and potassium, enhance or stunt plant growth and physiological processes, and contribute to regulating biomass allocation from vegetative growth to reproduction (e.g., Pigliucci et al. 1995; Yan et al. 2019; McDormand and Qaderi 2025). The joint influence of nutrient availability and temperature are found to have individual plant responses that scale to biogeographic patterns, such as water availability (Safavi‐Rizi et al. 2021; McDormand and Qaderi 2025), and emerge as essential components of global trends in morphological and physiological plasticity (Stotz et al. 2021; Reich and Oleksyn 2004). Combined effects of abiotic conditions may also reflect different plant responses than those to each environmental factor individually (Atkinson and Urwin 2012). Therefore a next logical step is to study quantitative traits throughout the lifespan and across multiple controlled manipulative environmental treatments to begin to make predictions on the influence of these individual gene regions in the context of multivariate complexity of phenotypic variation that exists in natural populations and environmental conditions.

To test these ideas experimentally from the perspectives of single‐gene mutation influences and from across development from seedling to reproductive fitness, we took advantage of the PhenoLeaf database containing qualitative size categorization of a collection of 18 day mutant seedlings from a reverse genetic screen (Wilson‐Sánchez et al. 2014). The mutants differ from Col‐0 genetically only by the single insertion mutation; nevertheless, a single insertion mutation has observable rosette differences in seedlings. Based on this published qualitative database, we built two sets of mutant lines: a set of mutants that produced a seedling with a larger rosette than wildtype (hereafter increase set), and a set of mutants that produced a seedling with a smaller rosette than wildtype (hereafter decrease set) in common culture conditions of temperature and light availability. Such robust variation based on a qualitative screen at such an early lifestage allows us to address an open question of whether the rank order of these two sets of mutants (increase and decrease sets) remained at later life history stages specifically rosette size at bolting (the onset of the flowering phase) and reproductive output. This investigation has the potential for uncovering functional influences of a single gene at multiple stages during the lifespan of the plant.

Growing mutants alongside populations representing natural variation contextualizes experimental investigations of single gene mutants in a common genetic background within the breadth of phenotypic variation found in nature (Rutter et al. 2019; Murren et al. 2020). Using this approach, Murren et al. (2020) demonstrated that some mutant lines in some nutrient conditions have phenotypic traits in root and fruit production outside the phenotypic expression of natural accessions. For the current examination, we employed a set of natural lines (also referred to as accessions or natural populations) that were obtained from Arabidopsis Biological Resource Center (ABRC). These genotypes were used as phytometers (Rutter et al. 2019). The phytometer concept dates back to Clements and Goldsmith (1924) where a specific group of plants in an experiment acts as a quantitative meter of the whole experiment's response to the environment. Using this experimental tool enables the relative scaling of traits across multiple growth chambers or experiments to a shared context. Additionally, in our experiment, we frame these responses in the context of natural variation among commonly studied accessions that differ genetically across many aspects of the genome. In this framework of prior qualitative data of seedlings from a reverse genetic screen, environmental treatment variation, and phytometer natural accession, we asked if mutant lines characterized by qualitative increased seedling rosette size, and those with decreased seedling rosette size maintained those predicted differences when temperature or nutrient conditions increased modestly as often occurs in nature. Alternatively, we investigated if one of the mutant sets benefitted or was harmed by environmental treatments in the common environmental growth chamber conditions. We hypothesized that the seedling rank order of increase and decrease seedling size sets would remain for rosette size at flowering time across environments. Consistent with this hypothesis, we expected to uncover parallel nutrient and temperature plastic responses. Additionally, we hypothesized that the set of mutant lines with increased rosette size from an early life qualitative screen would produce more fruit and the decrease set would produce fewer fruit; where the environmental conditions of warmer temperature would have higher overall fruit production for both of those sets, yet retaining the rank order of mutant sets as in benign temperature environments. As tradeoffs for flowering time, rosette size and overall reproductive effort can be phenotypically plastic, we tested whether the two mutant sets differed in tradeoffs within and across essential environments, with the expectation for a positive relationship between rosette size and fruit production in resource rich environments. While our goal was to explore the general responses of the sets of mutant lines and natural accessions, the experimental structure also allows for examination of within set variation among mutant genotypes to environmental treatments.

For a subset of these mutant lines with diverging responses to environmental variation, we investigated if photosynthesis modulates the response to temperature and nutrient conditions such that when rosettes are small, photosynthesis is enhanced to offset the reduced leaf area. Uncovering mutant lines that are distinct in whole plant form (rosette size and flowering) and distinct in CO_2_ assimilation acts as a means of providing mechanistic insight of gene influence and of physiological processes on morphological differentiation or tradeoffs. We hypothesized that the increased rosette mutant set would have greater CO_2_ assimilation and at warmer temperature would have greater CO_2_ assimilation, even when accounting for plant size. We also hypothesized that the decrease set at warmer temperature would have a decrease in CO_2_ assimilation and the opposite for the increase set. To test this series of hypotheses, we conducted manipulative experiments with Arabidopsis mutant lines and natural accessions grown across temperature and nutrient treatments to quantify traits throughout the life cycle and connect with reproductive fitness.

## 2 Methods

Wilson‐Sánchez et al. (2014) completed an extensive screen of 553 *Arabidopsis thaliana* Salk insertion mutants and built a database of qualitative rosette phenotypes (PhenoLeaf) on agar under continuous 24 h light at 20°C in growth chambers. Over 250 mutant lines were reported to have a relative rosette size shift from Col‐0 genetic control in these conditions, but less than 50 single‐insert mutants were identified to have increased relative seedling rosette size in the agar plate screen. From the detailed qualitative descriptions in their supplemental information, we subset mutant lines where substantial leaf qualitative responses were noted as “increased” in rosette size and “decreased” in rosette size from the Col‐0 wildtype (increase mutant set and decrease mutant set respectively) in seedlings 18 days after stratification. These two distinct sets thus represented a priori groups where the mutation either enhanced or decreased seedling rosette size through a single genetic mutation. We used these two categories, each with multiple mutant lines to represent distinct sets with the potential for enhanced or reduced rosette size at later life stages, serving as our formal groupings for testing our hypotheses. Due to the use of insertion mutant lines (O'Malley and Ecker 2010), and as the initial agar studies at the seedlings stage were conducted in the laboratory environment, the logical next step was to examine the full life cycle in controlled growth chamber conditions where abiotic variables can be easily manipulated.

The initial set of mutants we had identified went through additional filters. From the list of < 50 mutant lines, only some were available at stock centers (ABRC or Nottingham) and we obtained those available. Further, in a common growing environment, we bulked seed and conducted a molecular genetic approach to confirm that the each were single insert homozygous mutants. This PCR approach is described in Rutter et al. (2019). Briefly, the T‐DNA insertion and single‐copy reference gene were amplified using 2 distinct primer sets and the resultant PCR products were analyzed for relative intensities to determine the number of inserts. Following those steps, we had a set of 24 mutant lines that met all the above criteria; the resultant material available for experiments included 12 mutant lines in the decreased rosette size set and 12 mutant lines in the increased rosette size set. We used these 24 mutant lines to represent the two seedling rosette size sets in our quantitative studies of rosette size at the life history transition stage and reproductive fitness‐related traits. Further, this design would enable us to assess if the two sets differed in responses to environment, as well as characterize variation among mutant lines nested within sets of our planned comparison. In addition to mutant lines, wildtype (sensu Wilson‐Sánchez et al. 2014; Col‐0, stock center number CS70000) and 10 natural accessions which are a subset of those widely used in genomic studies (e.g., Exposito‐Alonso et al. 2019) with original seed collections from across Eurasia (see Supplemental of Rutter et al. (2019) Table S2 with metadata details of geographic origin) were bulked for use in the replicated experimental design. The natural accessions served as our phytometers. The planned use of the phytometers were as scalers to account for growth chamber microenvironmental variation (see below) and to contextualize phenotypes of mutants within natural variation. Col‐0 is the unmutated genetic control for the Salk insertion mutants used here and these mutants are all confirmed as single‐mutants within the Col‐0 parental background.

### 2.1 Trait Measurements

Individual plants were grown in separate pots in a replicated design across 4 growth chambers in a random block design. Seeds were cold‐treated in the dark for 1 week at 5°C on moist filter paper before individual seeds were germinated in pots on moist potting mix. Each growth chamber (GC, Percival, Ames IA) holds 432 plants spread across 6 trays each with 72 pots. GCs were set for a 16 h photoperiod. Two GC were set to 24°C and the other two GCs were set to 20°C such that growth chamber and temperature were independent, with plants treated with and without nutrients split evenly within each chamber and temperature. The temperature increase from 20°C to 24°C was previously shown to induce significant seed production differences in *A. thaliana* (e.g., Ibañez et al. 2017). Osmocote prills were added to modify the nutrient environment and each tray had one nutrient addition treatment and one no‐nutrient‐addition as replicate controls for each genotype. Col‐0 (ABRC Stock number COL70000) was replicated four times in each tray, two nutrients added and two control, no‐nutrient‐addition treatments. Each mutant line was replicated 12 times in each treatment, two times in each tray. One mutant, SALK_099684C, did not germinate in the experimental array and was removed from analysis described below. The full experiment included 1728 plants.

During the experiment, date of bolting was recorded, and rosette diameter at the day of bolting was recorded following the methods of Rutter et al. (2019). Briefly, date of bolting was recorded when the inflorescence was 5 mm in height, and the widest point of the rosette was measured with digital calipers. Mature plants were harvested when the main stem was no longer producing observable flowers. At harvest, the following traits were measured: fruit number (good fruit‐ those with seeds, and aborts‐ those flowers that did not result in fruit or did not produce seeds), and inflorescence height of the main stem (see Rutter et al. 2017, 2019, 2020 for further details).

### 2.2 Physiological Measurements

Following the initial growth chamber experiment, focal genotypes for the physiological measurements were chosen based on the initial reaction norms from plants grown in the temperature and nutrient experiment described above and represent a subset of those mutant lines phenotyped in Wilson‐Sánchez et al. (2014). This subset of mutant lines would enable us to characterize the breadth of potential responses and inform the broader question of whether a physiological mechanism could counter a physical reduction in leaf area. Our goal was not to be exhaustive in sampling, but rather to inform the capacity for such responses and amelioration from physiological processes. From each increase–decrease set, we chose two lines: one accession showing a clear decrease in rosette diameters from 20°C to 24°C, and the other accession showing a clear increase in rosette diameters from 20°C to 24°C. The accessions chosen from the decrease set were SALK_022843C and SALK_006410C respectively; the ones chosen from the increase set were SALK_022035C and SALK_096651C respectively. We selected only accessions that reflected their initial seedling rosette classification and ontology found in the PhenoLeaf database in our growth chamber experiment.

For physiological investigation of the chosen genotypes, seeds were germinated at 22°C. Upon germination, plants were transferred to a 20°C or 24°C chamber and were grown for 15 days in round pots for CO_2_ assimilation rate measurements using the Li‐6800 (LI‐COR Biosciences, Lincoln, NE) small plant chamber. An individual Col‐0, wildtype, was paired on each sample date with each mutant individual for direct comparison while minimizing micro‐environmental differences that can add variation to the physiological measurements if conducted on different dates.

Our approach was informed by Brazel et al. (2025), who demonstrated substantial differences between morning and afternoon physiological measures on the same Arabidopsis plant. Therefore, we restricted our measurements to the afternoon between 14:00 and 16:00 to standardize time of day. During each sampling window on each sampling date, we had paired Col‐0 and mutant. Each replicate was separately assayed for photosynthesis with plant present and for soil respiration by repeating the light response curve measurements on the empty pot. Plants were acclimated in the dark, in the small plant chamber for at least 5 min. The Li‐6800 was set to flow rate of 600 μmol s^−1^; sample water vapor set point at 19 mmol mol^−1^; reference CO_2_ set to 400 μmol^−1^; mixing fan speed at 10,000 rpm; chamber pressure at 0.2 kPa; light source composition at 90% red, 10% blue; using light intensity curve set points 0, 250, 500, 750, 1000 μmol m^−2^ s^−1^ with 120–180 s wait time between sets (following methods outlined in Brazel et al. 2025). This procedure was repeated for the mutant plant, the empty pot of the mutant plant and the wildtype and the empty pot of the wildtype. The entire experiment included 136 individual light response curve runs. We followed the methods of Brazel et al. (2025) to calculate carbon assimilation features taking into consideration soil respiration. We retained data only from plants where we had completed a full run of light response curves. Biologically implausible outputs derived from technical issues were removed (2 runs). All calculations are done using the maximum photosynthetic rate at high photosynthetically active radiation levels (*A*_max_). We accounted for differences in individual seedling size by calculating the leaf mass per area (LMA = plant mass/plant area). We used WinRhizo (Regent Instruments Inc., Canada) Arabidopsis version to calculate projected surface area of the intact rosette, and total leaf area (once leaves were dissected and scanned individually). Leaf mass was obtained on a Mettler Toledo microbalance XP6 (Columbus OH). Photosynthetic rate on a mass basis (*A*_mass_) was calculated by dividing the photosynthetic rate on an area basis (*A*_area_) by LMA (Wright et al. 2004).

### 2.3 Statistical Analyses

Data visualization and statistical analyses were performed with R (v4.3.1; R Core Team 2023) and R Studio (Posit Team 2023), using dplyr package (Wickham et al. 2023) for data management, ggplot2 (Wickham 2016) and ggpubr (Kassambara 2023) packages for data visualization. We ran a mixed model analysis of variance to test environmental and genetic components of our experimental design. We employed the packages lme4 (Bates et al. 2015) and lmerTest (Kuznetsova et al. 2017); using Satterthwaite approximations. The three sets of genotypes were categorized as the following: phytometers which included wildtype Col‐0 and natural accessions, increase mutants identified as larger rosettes as seedlings than wildtype, and decrease mutants identified as smaller rosettes as seedlings mutant in Wilson‐Sánchez et al. (2014), which we name as “line sets” hereafter. To leverage the phytometer variation and focus the study on biological variation in response to environmental treatments, statistical analyses and visualizations of our data are done with standardized values to account for growth chamber variation as a means to account for microenvironmental variation among replicate growth chambers, which although part of the experimental design was not the biological focus of our investigations. We calculate *z* values (*z* scores) following the methods of Rutter et al. (2019), where each data point is subtracted by the mean of phytometers by each chamber divided by the standard deviation of phytometers by chamber. We examined these fixed main effects and their pairwise interactions on rosette diameter, days to bolting, inflorescence height, fruit production and aborted fruit. Differences between the two mutant size sets would indicate that the early seedling rosette qualitative trait assessments were also observed in quantitative rosette size at the later developmental time at the onset of the reproductive period. Examination of differences among these sets and among mutant genotypes within sets performance across temperature and nutrients environments allows for the evaluation of new phenotypes to be discovered in distinct environmental growing conditions and the expression of phenotypic plasticity of flowering time, rosette size, inflorescence height and reproduction due to gene action at later life stages. We investigated genotype by environment responses to temperature and nutrients in two ways: among the sets (the phytometer natural accessions which included Col‐0, the increase rosette set, and the decrease rosette set) and among accessions (individual mutant lines or among phytometers) to understand if patterns of variation in responses at later life stages were consistent or differed across ecologically relevant environments, demonstrating genetic variation for plasticity. As each set was composed of multiple unique genotypes (10 phytometer accessions or 12 mutant lines per mutant set), we included a random nested effect of genotype within the set to account for the variation among genotypes. Contrasts were explored for significant fixed effects using the package emmeans (Lenth 2023) to examine using the Tukey method and Kenward‐Roger degrees of freedom. Finally, we investigated trait relationships between rosette size and flowering time, inflorescence height, aborted reproductive effort and total reproductive output. To investigate differences in tradeoffs (negative relationship) or positive association of rosette diameter and the other focal trait, we conducted similarity of slopes linear models using lm in base R (R Core Team 2023). We used likelihood ratio tests between full models and those without environmental treatment interactions with the line sets to investigate changes in slope of the relationship between rosette size and our other traits between line sets and across our temperature and nutrient environments.

## 3 Results

### 3.1 Rosette Mutants, Temperature, Nutrients

**FIGURE 1:**
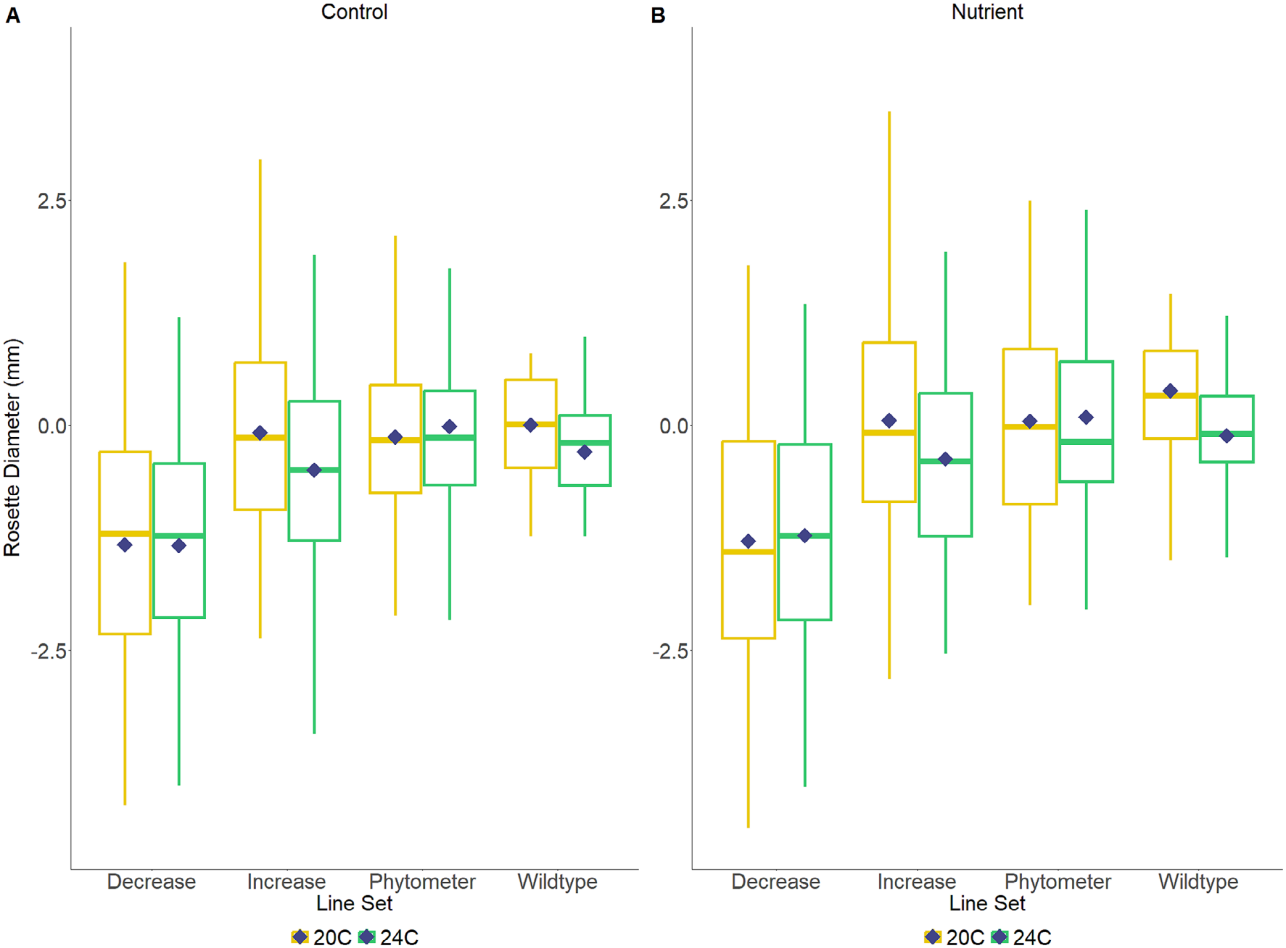
Sets of single insert mutant lines in the Col‐0 background identified by Wilson‐Sánchez et al. (2014) as decrease in rosette size, increase in rosette size in comparison to the wildtype Col‐0 as 18 day old seedlings. Phytometers are a set of natural accessions used as experimental scalers. Box plots are for standardized (*z* values) for rosette diameter at bolting which is the transition to the reproductive stage. Boxplots for temperature treatments are indicated by colors 20°C (yellow) and 24°C (green). Panels are the (A) control and (B) nutrient addition treatments. Box features include median as the horizontal line, the 25th–75th percentiles or interquartile range as the box, the interquartile range above the 75th or below the 25th percentile as the vertical lines, outliers as points, rhombus as mean values.

**FIGURE 2:**
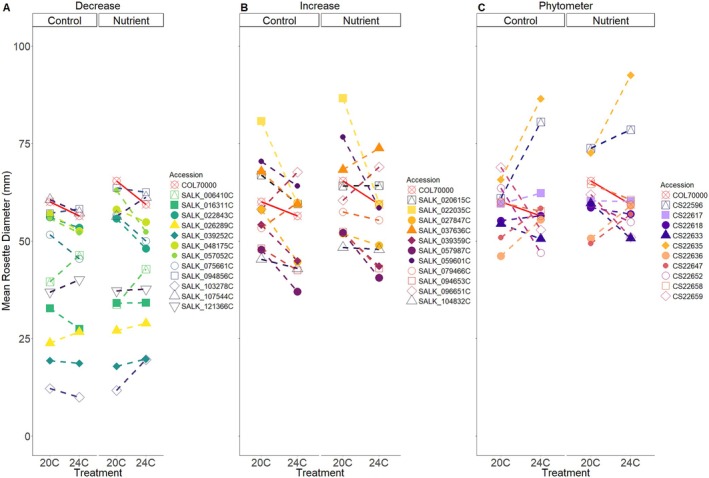
Mean rosette diameters at bolting (mm) reaction norms of each mutant and natural accession genotype. Lines connect trait means per genotype at 20°C and 24°C. Paired panels are for the control and nutrient addition treatments. Line sets are as defined in Figure 1, (A) decrease (B) increase (C) phytometer line sets. Individual genotypes are indicated by shape and color. Each panel contains the wildtype (Col‐0, as COL70000 here; solid line, open red circle) across the temperature and nutrient treatments. Wildtype is plotted in each of the panels for ease of comparison.

We detected significant rosette diameter differences overall between the three sets of genotypes (Figure 1; Table 1; *F*_2,30.96_ = 7.63, *p* < 0.002) when the growth chamber effects are accounted for by using *z* scores. The decrease mutants were significantly smaller than the natural accessions and wildtype (Figure 1; df = 31, *t* = 3.63, *p* = 0.028), and significantly smaller than the increase mutants (Figure 1; df = 31, *t* = −3.005, *p* = 0.014). For rosette size at onset of flowering, the rank order from the qualitative early seedling rosette screen for size was maintained for the decrease mutants. These general patterns are also obvious when observed by average for each individual mutant line (Figure 2). Viewed on the measured trait scale in Figure 2, a majority of the decrease set have smaller rosette diameters compared to the wildtype across all temperature and nutrient treatments.

**TABLE 1:** Effects of line set (increase, decrease insertion mutant lines defined by Wilson‐Sánchez et al. (2014) based on qualitative size at 18 day old seedlings in comparison to the genetic background wildtype Col‐0 and wildtype/phytometer, which are natural accession used as scalers and for ecological context), temperature treatment (20°C, 24°C), nutrient treatment (control, nutrient addition) and pairwise interactions for our focal traits of rosette diameter, fruit production, and aborted fruit production. Individual genotypes (a.k.a. accessions) are nested within line set in this linear mixed model. Statistically significant factors are in bold values.

Mixed models	Factor	Numerator df	Rosette diameter			Fruits			Aborted fruits			Aborted/total fruits		
			Denominator df	*F*	*p*	Denominator df	*F*	*p*	Denominator df	*F*	*p*	Denominator df	*F*	*p*
Type 3 ANOVA Satterthwaite	Line set	2	30.96	7.6314	**0.002021**	29.76	7.1322	**0.002948**	30.91	2.9391	0.067837	30.33	5.7594	**0.007581**
	Temperature	1	1627.97	22.7482	**2.01E−06**	1564.01	18.2594	**2.04E−05**	1621.94	17.1445	**3.64E−05**	1564.62	8.7699	**0.003109**
	Nutrient	1	1627.97	9.5147	**0.002073**	1563.83	38.0488	**8.77E−10**	1621.94	84.9398	**2.20E−16**	1564.44	38.9251	**5.66E−10**
	Temperature × Nutrient	1	1627.97	0.082	0.774663	1563.77	3.8589	**0.04966**	1621.93	7.3802	**0.006665**	1564.37	10.2842	**0.001369**
	Line set × Temperature	2	1627.97	13.4352	**1.63E−06**	1564.01	4.0749	**0.017175**	1621.94	14.7079	**4.67E−07**	1564.61	15.0404	**3.39E−07**
	Line set × Nutrient	2	1627.97	0.2333	0.791957	1563.83	3.6592	**0.025974**	1621.94	0.5306	0.588328	1564.44	0.1086	0.897088
			**df**	**LRT**	***p***		**LRT**	***p***		**LRT**	***p***		**LRT**	***p***
Random Effect	Accession: line set		1	955.47	**2.20E−16**		354.35	**2.20E−16**		427.26	**2.20E−16**		412.26	**2.20E−16**

When examining the manipulated environmental treatments, rosette diameter was significantly larger overall in 20°C than 24°C (Figure 1; Table 1; *F*_1,1627.97_ = 22.75, *p* < 0.0001), and in the added nutrients than the control (Figure 1; Table 1; *F*_1,1627.97_ = 9.514, *p* = 0.0021). Additionally, temperature and line set factors had interaction effects on the rosette size: larger rosette diameters at a lower temperature (20°C) that were significantly more pronounced in the increase set (Figure 1; Table 1; *F*_2,1627.97_ = 13.4352, *p* < 0.0001). We found significant genetic variation among mutant lines as detected from a random effect of individual genotypes within sets in the analysis of variance (Table 1; LRT = 995.47, df = 1, *p* < 0.0001). While overall the increase set rosette at bolting was significantly larger than the decrease set, not all individual mutant lines within that set were larger than wildtype (Figure 2, visualized on the measured trait scale). Of the 11 lines, only four mutants of the increase mutant set had larger rosette diameters compared to the wildtype in each of the temperature and nutrient treatments (Figure 2). Of particular note is the SALK_026289C mutant line (Figure 2; yellow triangle), which was one of the extremely small mutants outside the natural variation of phytometers and even among the decrease set.

### 3.2 Rosette Size Mutants Reproductive Fitness Across Environments

**FIGURE 3:**
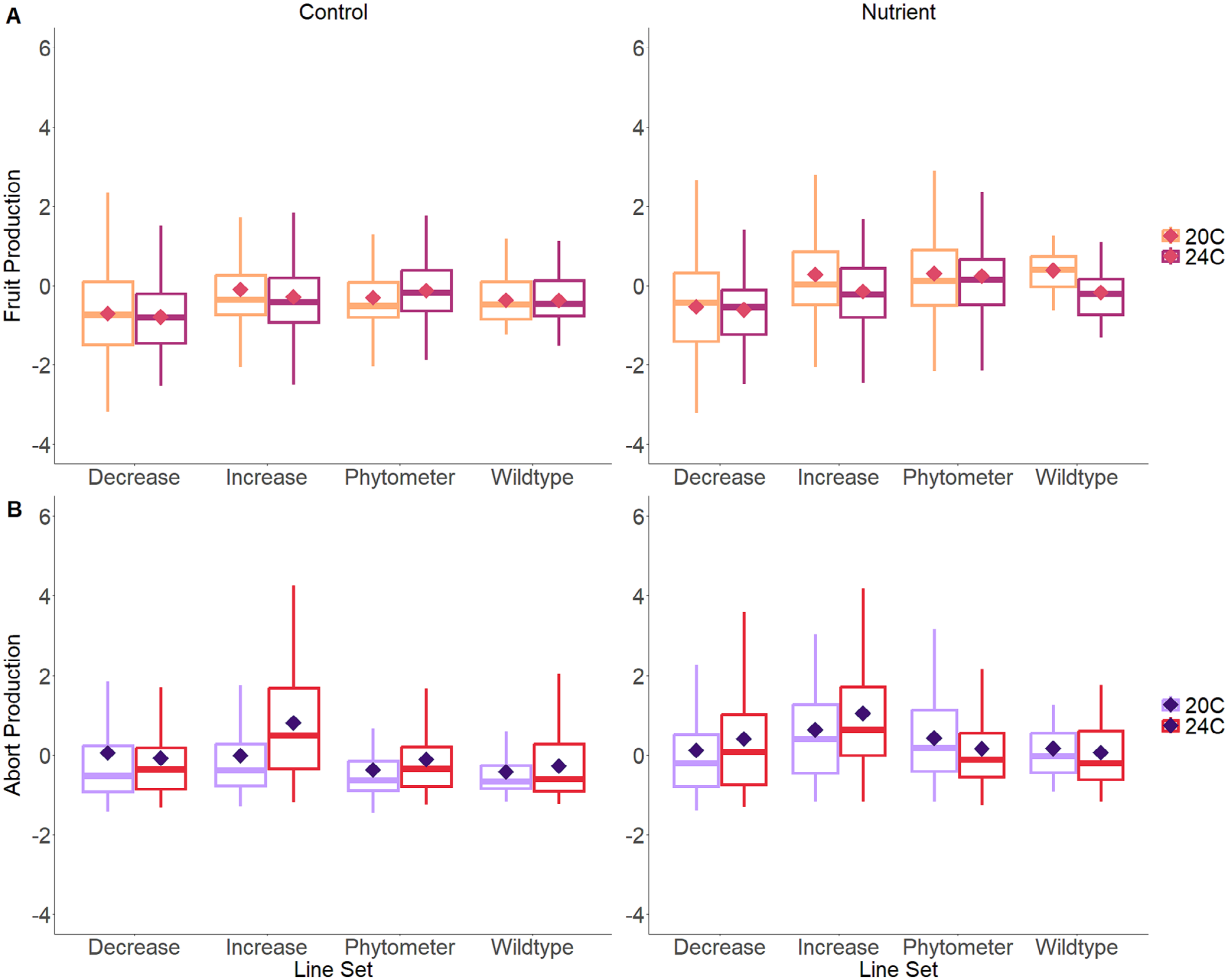
(A) Box plots of increase, decrease, phytometer, and wildtype sets as defined in Figure 1, are plotted for standardized fruit production (*z* values). Control (left) and nutrient addition treatments (right). Temperature treatments are indicated by colors 20°C (orange) 24°C (magenta). Boxplot features are as above. (B) Box plots of increase, decrease, phytometer, and wildtype sets are plotted for standardized aborted production (*z* values), defined as the reproductive effort that did not result in seed production. Control (left) and nutrient addition (right). Temperatures are indicated by colors 20°C (pale purple) 24°C (red). Boxplot features are as above.

We detected significant differences across sets of genotypes for fruit production (Figure 3A; Table 1; *F*_2,29.76_ = 7.13, *p* = 0.002), with the decrease set being lower than other sets (Figure 3A; df = 31, *t* = 3.365, *p* < 0.006) and significantly lower than the increase set (Figure 3A; df = 31.1, *t* = −3.121, *p* < 0.01). Nutrient treatments and their interaction with temperature significantly influenced fruit production (Figure 3A; Table 1; nutrients: *F*_1,1563.83_ = 38.05, *p* < 0.0001, nutrient × temperature: *F*_1,1563.77_ = 3.86, *p* = 0.0496), with greater fruit production in the case of nutrient addition and with nutrient addition at lower temperatures. Nutrient addition significantly increased overall inflorescence height (Figure S1; Table S1; *F*_1,1621.97_ = 13.34, *p* < 0.001) as did the warmer temperature (24°C) (Figure S1; Table S1; *F*_1,1621.97_ = 28.11, *p* < 0.001). The three sets of genotypes (line sets: the natural accessions and two mutant sets) differed in response to temperature and nutrient treatments for fruit production (Figure 2A; Table 1; line set × temperature: *F*_2,1564.01_ = 4.07, *p* < 0.02; line set × nutrient: *F*_2,1563.83_ = 3.66, *p* < 0.03). We detected significant variation among individual lines (Figure 3A; df = 1, LRT = 354.35, *p* < 0.0001) within each of the three line sets (increase, decrease or phytometer accessions). Temperature and line set factors had interaction effects on inflorescence height: taller inflorescence at the warmer temperature that is more pronounced in the increase set (Figure S1; Table S1; *F*_2,1621.97_ = 4.996, *p* < 0.01).

**FIGURE 4:**
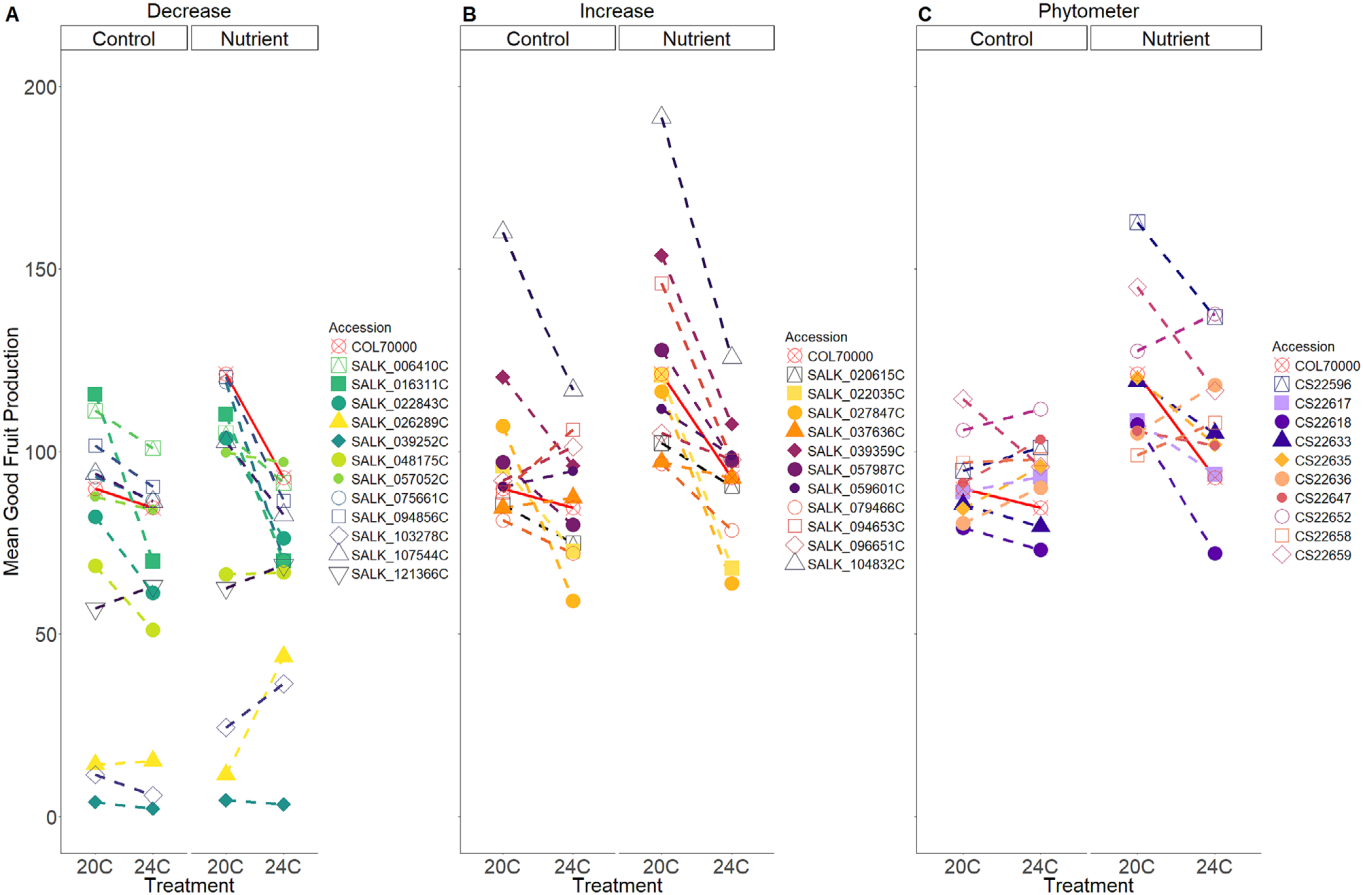
Mean good fruit (fruit that produced seeds) reaction norms of each genotype across 20°C and 24°C temperature treatments. Paired panels are the control and nutrient addition soil treatments. The line sets are as defined in Figure 1: (A) decrease (B) increase (C) phytometer line sets. Panel features are as above.

Overall, decrease mutants had lower fruit production compared to the wildtype, especially with the addition of nutrients (Figure 4, visualized on the measured trait scale). Additionally, the decrease set had smaller fruit lengths, more noticeable at lower temperatures across all the sections of main stem (Figure S2). The majority of the increase mutants have greater or similar fruit production compared to the decrease set. From 20°C to 24°C, a majority of the accessions show a general decrease in fruit production, a pattern that was more pronounced in the nutrient addition treatment (Figure 4).

**FIGURE 5:**
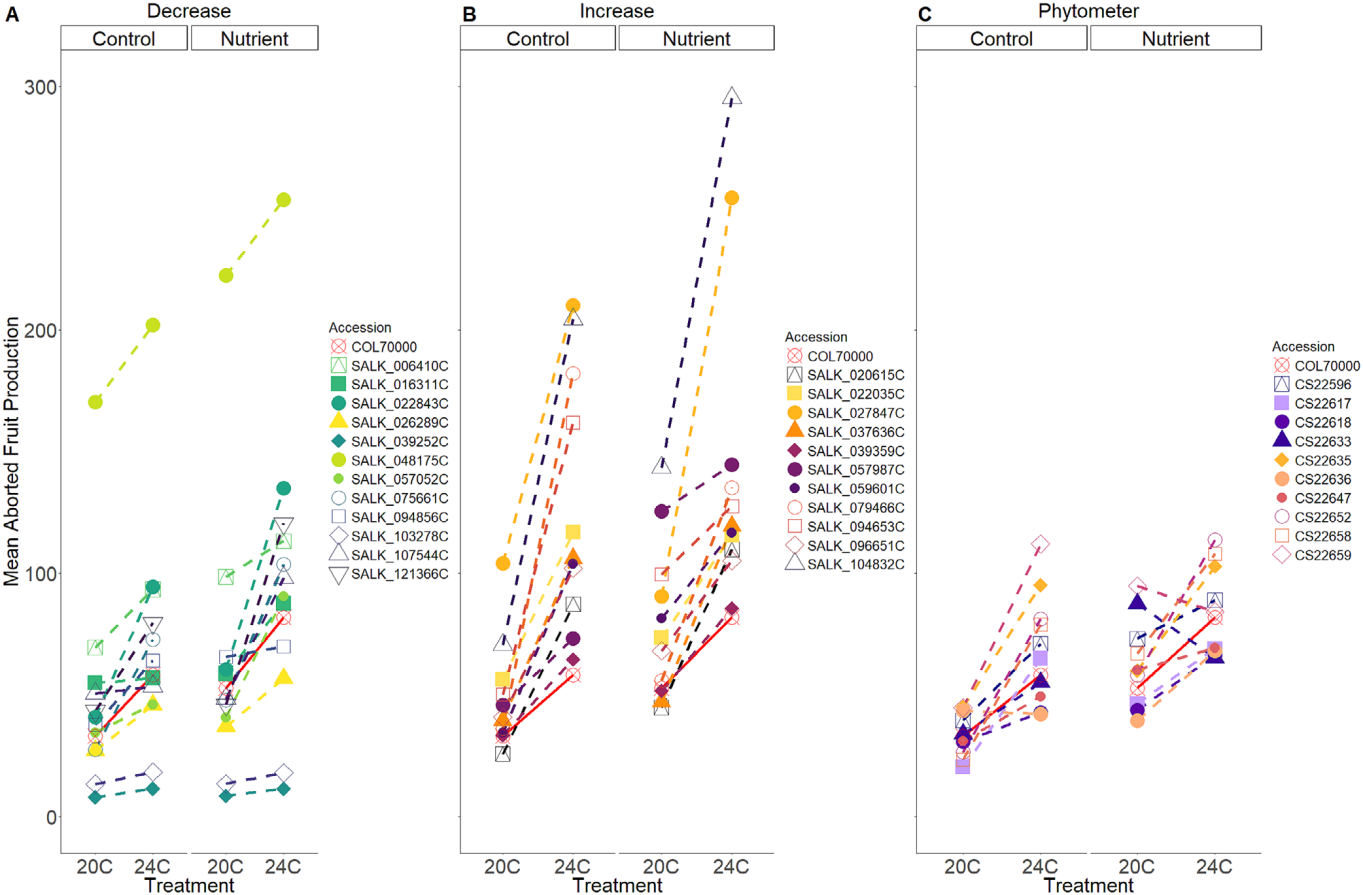
Mean aborted fruit production (reproductive effort not resulting in seed set) reaction norms of each genotype at 20°C and 24°C, with paired panels for control and nutrient addition treatments. The panels are defined as in Figure 1: (A) decrease (B) increase (C) phytometer line sets. Other panel features are as above.

There was overall significantly more fruit abortion at higher nutrients (Figure 3B; Table S1; *F*_1,1621.94_ = 84.94, *p* < 0.001), and at the warmer temperature (Figure 3B; Table 1; *F*_1,1621.94_ = 17.14, *p* < 0.0001). We found a significant interaction between these abiotic treatment types (Figure 3B; Table 1; *F*_1,1621.93_ = 7.38, *p* < 0.007). Increase mutants showed a pattern of higher fruit abortion compared to the decrease and natural accession sets across all the temperature and nutrient treatments (Figure 3B; Table 1; *F*_2,30.91_ = 2.94, *p* < 0.07). Temperature and line set factors significantly interact with fruit abortion: the increase mutants at the warmer temperature (24°C) had higher fruit abortion compared to the decrease set (Figure 3B; Table 1; *F*_1,1621.94_ = 14.7, *p* < 0.0001). We also detected significant differences in rosette diameter and fruit abortion slope between increase and decrease sets at 24°C (Figure S3; *p* < 0.0001). Most of the accessions in the decrease set showed higher fruit abortion compared to wildtype. SALK_048175C specifically has extremely high mean fruit abortion (Figure 5, visualized on the measured trait scale). Almost all the increase mutants show higher fruit abortion compared to wildtype at 24°C in both nutrient treatments (Figure 5). A majority of the accessions show an increase in fruit abortion from 20°C to 24°C (Figure 5).

**FIGURE 6:**
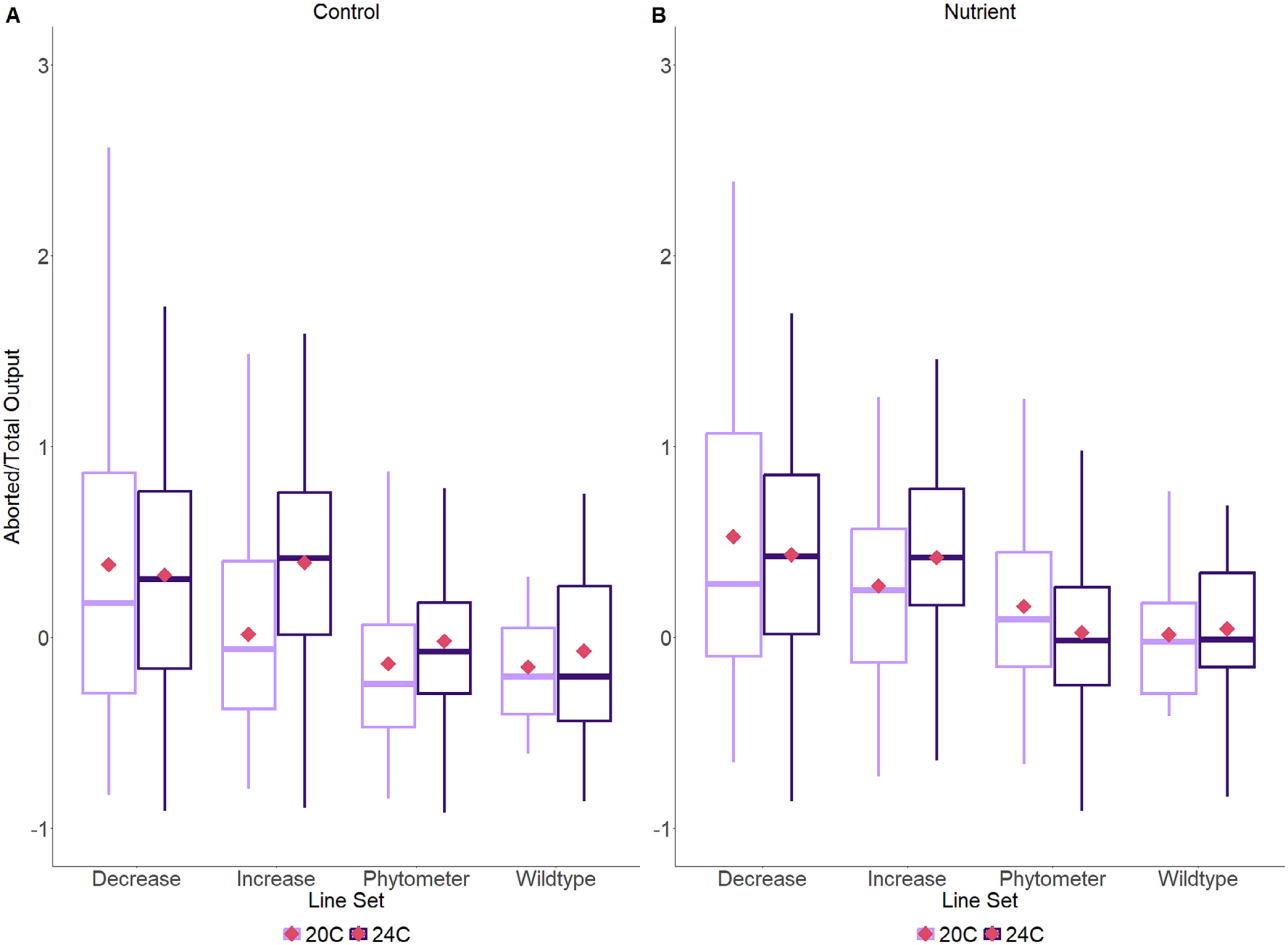
Box plots of increase, decrease, phytometer, and wildtype sets (as defined in Figure 1) are plotted for standardized aborted fruits divided by total reproductive output (*z* values) paired by temperature treatments. Panels are (A) control and (B) nutrient addition treatments. Box features include median as the horizontal line, the 25th–75th percentiles or interquartile range as the box, the interquartile range above the 75th or below the 25th percentile as the vertical lines, outliers as points, rhombus as mean values.

Line sets had significant differences in the proportion of aborted fruits produced to total reproductive output (Figure 6; *F*_2_ = 5.759, *p* < 0.01). We detected significant differences in aborted fruit proportion across the temperature and nutrient treatments (Figure 6; Table 1; temperature: *F*_1,1564.62_ = 8.769, *p* < 0.001; nutrient: *F*_1,1564.44_ = 38.92, *p* < 0.001). Temperature and line set factors did significantly interact: decrease mutant set shows an overall high proportion of aborted fruit produced to total reproductive output compared to the increase mutants, a pattern that was more pronounced at the warmer temperature (Figure 6; Table 1; *F*_2,1564.61_ = 15.04, *p* < 0.001).

### 3.3 Trait Relationships in Leaf Mutants Across Environments

We examined the relationship between rosette diameter, flowering time, inflorescence height, and reproductive output. Across our controlled abiotic conditions, we detected significant differences in slope for rosette diameter and fruit production between increase and decrease sets (Figure S4; *p* < 0.0001). Along with smaller rosettes, the decrease set also has shorter inflorescences compared to the wildtype (Figure S1; df = 31, *t* = 2.799, *p* < 0.05). We detected that while rosette size, inflorescence size and fruit production were positively associated (Figures S4 and S5) there were significant differences in the relationship between mutant sets and across temperatures (Table S1). The relationship between rosette diameter and inflorescence height for the increase mutants is different from the decrease set (Figure 5; *p* < 0.0001). Of particular note is the relationship between rosette size and days to the onset of the reproductive phase (days to bolting). The decrease mutants show the tradeoff between rosette size and days to bolting, with smaller rosettes correlating with delayed onset of reproduction (Figure S6). This pattern is flipped from tradeoff to a positive association in the phytometers and wildtype at 24°C. There are significant differences across line sets for days to bolting (Figure S7; Table S1; *F*_2,30.96_ = 4.629, *p* < 0.05), with the increase mutants showing overall faster reproductive timing than the decrease set (Figure S7; df = 31, *t* = 2.957, *p* < 0.05). While increase mutants generally have larger rosettes than decrease mutants (Figure 1), the association between rosette size and reproductive output is weaker for this set in both temperature treatments. Both increase and decrease sets have a positive association with rosette diameter and inflorescence height.

### 3.4 Plasticity of CO_2_ Assimilation for Representative Leaf Mutants

**FIGURE 7:**
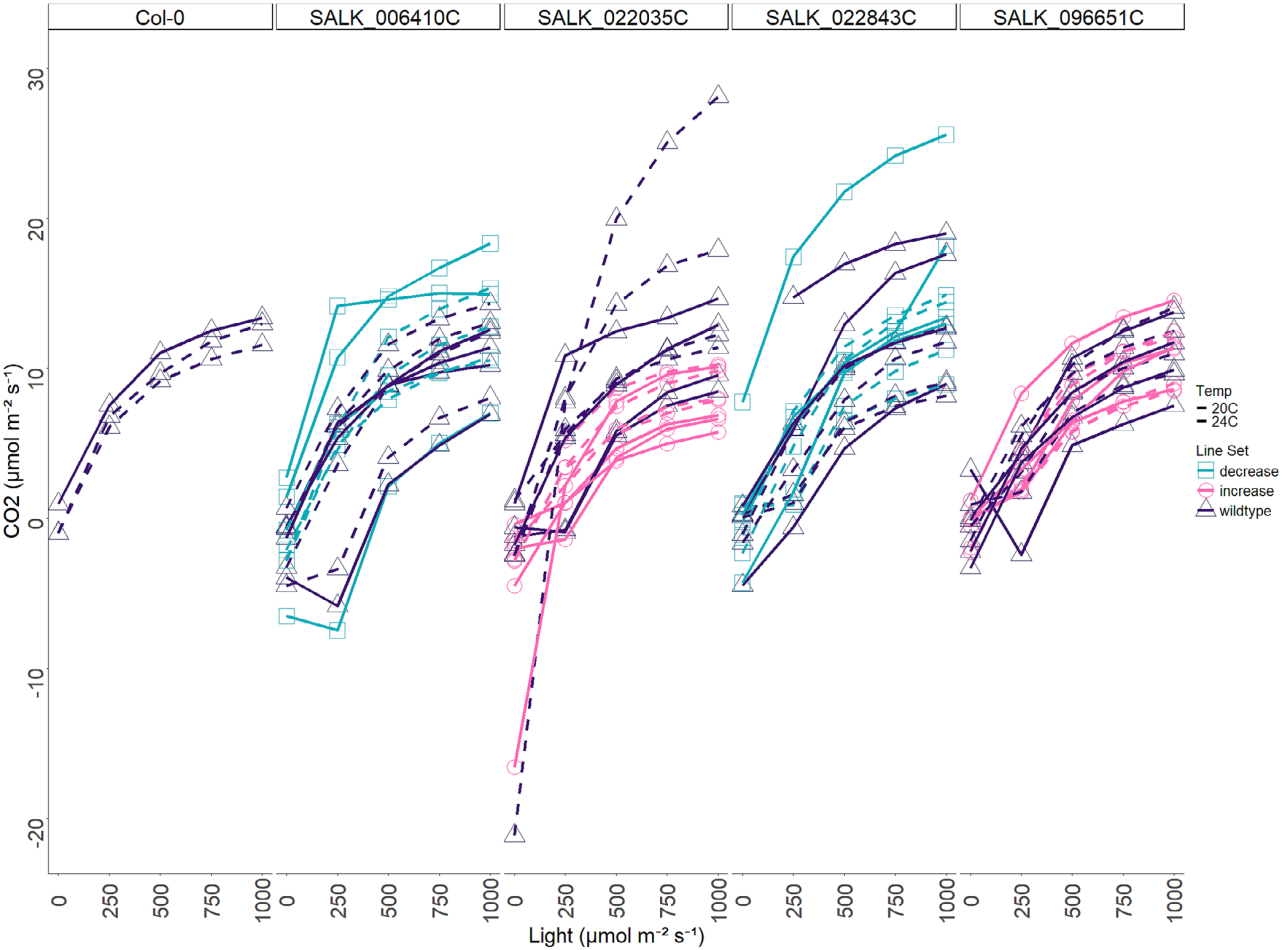
Projected surface leaf area adjusted gas exchange light response curves for the 5 focal genotypes: SALK_006410C, SALK_022035C, SALK_022843C, SALK_096651C. Each mutant replicate is paired with a wildtype (Col‐0) grown under the same environmental conditions. Additional replicates of the wildtype are plotted alone. The projected leaf area used for adjustment is based on the entire rosette. The dashed line represents plants grown in 24°C treatment and the solid line represents plants grown in 20°C treatment. Blue squares are lines in the decrease set, pink circles are lines in the increase set, and dark triangles are in the wildtype set (Col‐0). CO_2_ assimilation is ordered from increasing light intensities from 0, 250, 500, 750, 1000.

Across temperatures, decrease mutants have smaller rosette areas compared to the increase mutants (Figure S8). Notably, the increase line, SALK_022035C, had an average of larger rosette area of seedlings used in the physiological measurements compared to wildtype in 24°C (Figure S8). Despite each line showing different morphological size responses to initial temperature treatments (Figure S9) and with distinct size differences in rosette area, decrease and increase sets, light response curves were generally similar (Figure 7; Figures S10 and S11) despite the distinct leaf areas (Figure S8). When overlayed together, in both temperatures, the decrease mutants trend with higher *A*_max_ than increase mutants; however, when taken in context with the variation of the wildtype, the two sets are not distinct (Figure S12). Since the selected mutants were morphologically plastic lines with a greater chance of demonstrating distinct photosynthetic curves based on quantified morphological differences, other lines within the sets may reflect similar curves. While the two mutant sets were generally similar, we detected significant differences among accessions in *A*_max_ (Figure 7; *F*_4_ = 4.690, *p* < 0.05). Wildtype light curves were quite varied, notably at 24°C within the SALK_022035C group (Figure 7).

## 4 Discussion

Characterizing phenotypic variation in two sets of Arabidopsis mutant lines previously identified to have *qualitative* differences as 18 day old seedlings, with *quantitative* measures of rosette size at the onset of the reproductive phase provides evidence for genetic and environmental influences on reaction norm variation based on a single mutation. We set out to test the notion that individual gene effects on rosette size could result in shifting tradeoffs between vegetative and reproductive traits, which may be distinct across ecologically relevant abiotic environmental treatments. Our study demonstrates that functional effects of mutations are context dependent and quantitative evidence shows that the characterization of mutants at early seedling stages does not necessarily reflect later life stage rosette measures, whereby genetic and environmental interactions shape phenotypic traits throughout the entire lifespan. Our findings within environmental treatments are consistent with our hypothesis of maintaining rank order between seedling and later rosette size for the decrease set, but the increase set does not consistently exceed the wildtype (Col‐0), indicating that early seedling classifications are not uniform predictors of later rosette size.

Our study underscores the importance of environmental context for studying single‐gene influences on morphological phenotypes, and cautions that patterns considered as broadly understood (Mitchell‐Olds 1996; Baker et al. 2005; Ellis et al. 2021) are in fact not necessarily uniformly distributed across genetic variants or ecologically relevant environments. Across 20°C and 24°C and nutrient addition, single insert mutants showed non‐parallel reaction norms for rosette diameter and reproductive traits. Importantly, we found that mutants categorized by qualitative size show fitness patterns that are abiotic treatment context dependent, with the set of mutants showing enhanced responses in reproductive traits to temperature and nutrient treatments, while rosette trait differences between line sets did not significantly change in nutrient treatments. Overall, reproductive output matched our prediction for the decrease set, but as observed in rosette size at bolting, our increase set did not uniformly outperform wildtype, reinforcing substantial variation within sets established by qualitative categorization. Mutants that differ in just a single insertion mutation reflect a wide range of responses to environmental treatments, demonstrating that a single gene that influences 18 day old seedling size results in remarkable intra‐specific variation at later life stages, and the mutation of a single seedling‐leaf associated gene disrupts the relationship between vegetative and fitness traits. In some cases such variation exceeds that observed in a set of natural accessions which represent geographic ecological origins and latitudinal variation ranging from the UK to Tadjikisatan and from Spain to Norway (Rutter et al. 2019), indicating a single mutation in a common genetic background can produce distinct phenotypic variation, and in other cases fall within observed, natural variation. We intentionally studied a subset of lines for light response curves that differed in morphological reaction norms. Interestingly, despite rosette size differences, light response curves, our contrasting mutant sets were found to be generally similar within and across temperature treatments, suggesting a decoupling of photosynthetic mechanisms and leaf size. Our selection of a subset of mutant lines with distinct morphological reaction norms was intentional to offer the greatest chance for exposing the potential of physiology to offset smaller rosette areas. Even with this strategic sampling, such physiological distinctions between increase and decrease mutant sets were subtle, and abiotic contexts outweighed differences in mutants for physiological traits.

### 4.1 Temperature and Nutrient

Development of leaf size is controlled by numerous genetic factors (Horiguchi et al. 2005) and determinants of leaf size such as maximum relative cell proliferation rate and primordium cell number are highly influenced by genetics, growth conditions or a combination (Ma et al. 2025). Our increase mutants could be constrained by cell size or growth tradeoffs that limit extremely large rosette sizes (Pigliucci and Hayden 2001), which may have been more evident at the stage we measured when plants are transitioning to reproduction and given time to grow to full size than in seedlings. The tradeoffs between vegetative growth and reproduction offer insights into the allocation of plant resources transitioning from the developmental phase to reproduction (Weiner 2004). For example, evidence supports sexual allocation and flowering time as linked traits that are inherent in plant growth and reproduction, with earlier flowering correlating with smaller plants and less reproductive biomass (Baker et al. 2005). While that analysis focused on genetic loci specifically involved in flowering time (Baker et al. 2005), our study focused on whether genes related to seedling vegetative size still support the same reproductive tradeoffs. Our experiment uncovered that increase mutants bolted earlier at the warmer temperature than wildtype, and with our observation that a majority of our increase mutants were not larger than wildtype as we expected, and the decrease mutants produced distinctly fewer fruit compared to the increase mutants. These patterns support the notion that growth and reproductive traits are fundamentally linked and single genes affecting vegetative size can have pleiotropic effects on reproduction. Increase mutants may, as they approach reproductive size threshold conditions required to bolt, instead of allocating resources to an increased rosette size, allocate resources to the production of more fruit or flowers (Baker et al. 2005). Decrease mutants also bolted earlier at warmer temperatures, with smaller rosettes than wildtype, but still had delayed bolting compared to wildtype. Single genes related to smaller rosette sizes suggest that while delayed flowering might be associated with larger rosettes, genetic constraints are still fundamental factors that influence trait relationships. These findings add to current literature that growth and reproductive traits are in fact abiotic treatment context dependent and here we specify even those affected by a single gene mutation. Reaction norms were not parallel among our pre‐described sets of mutant lines as they both responded to nutrients and temperature, but the magnitude and direction varied. This pattern is consistent with genotype by environment interactions, particularly with substantial variation among lines within each set. We also amplify that environmental context is an important consideration and broad patterns previously understood descriptive patterns from the literature are not necessarily uniform across all genetic variants.

Contrary to our prediction of warmer temperatures increasing successful fruit, we detected increased fruit abortion and reduction in successful seed producing fruit, particularly in the increase set. Additionally, as expected rosette size at bolting and fruit production were positively related, but the strength of the relationship differed by set and temperature, indicating that single gene mutations can alter the slope of the vegetative‐reproductive relationships, in addition to shifting mean trait values. Environmental conditions can play significant roles in ovule and bud abortion (Sun et al. 2004; Warner and Erwin 2005) where support for increased exposure and duration of flowers to substantially increased (33°C) temperature exacerbates floral bud abortion. Our temperature study was not as extreme. We focused on a temperature treatment warm enough to elicit a quantitatively detectable phenotypic response that could be the difference between warmer or cooler years in global climate (IPCC 2021), and our mutants were observed to have greater fruit production in lower temperature with nutrient addition and greater fruit aborted in the our warmer temperature condition. Since our plants were subjected to the temperature treatments for the entire period of the experiment, our findings suggest that prolonged warm temperature exposure still contributed to an increased fruit abortion, and consequently fewer fruit that produced seed, in the increase set, which additionally may have a greater temperature sensitivity than the decrease set. Osmotic conditions and temperature can also alter resource transport to floral ovaries (Weiner et al. 1997; Sun et al. 2004), so despite having sufficient nutrients available, reproductive demands may exceed nutrient transport to developing ovules, increasing fruit abortion. Interestingly, plants also responded to higher nutrient levels with an increase in aborted fruits in both temperature treatments, with a higher proportion of the total fruits being aborted as well. While nutrients may not be utilized effectively, that does not explain why in higher nutrient conditions, plants experienced an overall greater fruit abortion. Weiner et al. (1997) found that plants generally produce more seeds with excess resource availability rather than higher quality seeds (mass). More generally, under increased nutrient resources, fruit and seed production can be limited by resource partitioning and remobilization, particularly during senescence (e.g., Masclaux‐Daubresse et al. 2010; Havé et al. 2017). Plants in nutrient addition treatment could be initiating more flowers than resource transport can be utilized at higher nutrient levels, leading to increased number of flowers that do not result in successful fruit.

### 4.2 Leaf Area and Light Response Curves

Despite having overall smaller rosette size, decrease mutants were able to maintain photosynthetic rates similar to wildtype and increase mutants. Photosynthetic measures did not differ consistently between increase and decrease sets across temperatures, suggesting that this physiological insight does not fully explain why smaller rosettes can maintain comparable reproductive output. Our observations follow Warner and Erwin's (2005) experimental data, where photosynthetic responses of distinct accessions were similar before and after exposures to warm temperature. Ma et al. (2025) suggests that other cell features have an influence on photosynthetic assimilation rate that leaf area evolves independently, allowing plants to have varied leaf sizes without compromising high photosynthetic rates, thus vegetative size alone may not predict the capacity for carbon acquisition. For certain Arabidopsis genotypes, inflorescence photosynthesis can contribute a substantial amount of carbon for maintaining reproduction, and are able to produce high numbers of seeds even with earlier flowering (Earley et al. 2009; Gnan et al. 2017), which was not included in the measurements that we present here. However, inflorescence physiology may add additional insights to mechanisms on how smaller rosettes were able to maintain good fruit production similar to wildtype levels and how earlier flowering in the increase mutants did not significantly compromise good fruit production. Additionally, inherent in cue‐based physiological responses of leaves is phenotypic plasticity and the maintenance of the efficiency for light absorption and capacity for CO_2_ assimilation. These traits are linked to the genetic variation of transcription factors contributing to capacity for environmental responses (Hüner et al. 2013). Decrease mutants with smaller rosette size might have greater leaf thickness or other morphological and physiological traits that allows for the maintenance of photosynthetic efficiency despite having smaller leaf areas compared to the set of increase mutants. At lower temperatures, fast growing species have higher net assimilation rate due to reduced carbon used in respiration (Loveys et al. 2002). However, our results did not reflect this general pattern at 20°C and may require even cooler temperatures to be evident (e.g., Loveys et al. 2002 plants were grown at 18°C). Perhaps with a lower temperature, greater differences in assimilation rates for our mutants would have been detected, however within our experimental temperature range, the mutants did not respond differently from wildtype, showing that mutations that influence rosette size do not necessarily affect photosynthetic capacity and that photosynthetic components are more decoupled. As we had observed that with warmer temperatures, fruit abortion was significantly higher for both mutant sets may also be related to physiological considerations. Photorespiration also increases with increasing temperatures, reducing overall carbohydrate yield (Ferguson et al. 2021) and warmer temperatures increases plant respiration, which may lead to faster depletion of carbohydrates required for fruit development (Loveys et al. 2002).

One limitation in our photosynthesis data is that we observed a couple of measurements that were extremely negative in the SALK_022035C group. For that sample, the relative humidity (RH) of the plant in the chamber was significantly lower than all other plants measured. Additionally, differences in CO_2_ flux versus water flux was noted, where an imbalance of excess water compared to CO_2_ negatively affects the ability of the dilution correction factor in the assimilation equation to compensate for the greater volume of water in the air than CO_2_. These technical limitations in part may be what we observed in those two extreme light response curves for these individuals. Such patterns lead us to recommend, when feasible, in the future experiments to enhance the amount of replicates. Due to the complex logistics of collecting light response curves for CO_2_ assimilation, sample sizes for assimilation curves are constrained by time available with the instrument. Therefore we selected mutants with divergent phenotypes to explore if the extremes in reaction norms were buffered by physiology. Such patterns uncovered context dependency of morphological and physiological responses as seen in other species to nutrients and water availability in growth chamber experiments (e.g., Safavi‐Rizi et al. 2021). With these results on physiological and morphological traits in growth chambers, appropriate future confirmation with field data would be appropriate next experiments to further the context of climate change. We found overall that despite divergent morphological differences, photosynthetic curves remained close to wildtype. Our data do not suggest that for early seedling leaf mutants of distinct size that the photosynthetic rates are driving these patterns of divergence. Thus, studying CO_2_ assimilation for more mutant lines with a focused analysis in particular genetic pathways in response to temperature and nutrients could provide a distinct look at these responses yet leveraging single gene mutant sets to fill in the details of gaps from another complementary perspective.

### 4.3 Examples of Gene Function Context

Our explicit goal in this study was to contrast the full life cycle and reproductive responses of sets of single gene mutations with distinct rosette size actions in 18 day old seedlings, in the context of modestly warmer temperatures and modestly enhanced nutrients as might commonly be found in nature across local field sites or between years. While the contrast of sets of mutant genotypes that represent increased or decreased seedling size for fitness was our explicit goal, in tandem, we can also begin to explore the functional relationship of some individual genes where prior information is available from other studies. For example, SALK_026289C has an insertion in a gene influencing a plastid ribosomal protein (Table S2), with the At2g24090 locus being characterized as essential for fitness‐related traits of seed and embryonic development (Romani et al. 2012). The irregular cell division patterns from disruptions in plastid translation likely contributed to cascading effects through development of the extreme decreased rosette size and low fruit production that we observed. The SALK_022035C line has an insertion influencing phytochrome B (Table S2), and genetic disruptions to phytochrome B have been shown to impact signaling pathways involved in regulating de‐etiolation and seedling sensitivity to red light (Zhang et al. 2013). Mutation resulting in the disruption to the sensitivity of red light in phytochrome B could have affected the regulation of petiole elongation for this line, increasing total width of the plant (Zhang et al. 2013). While phytochrome B has influences on photosynthesis (Boccalandro et al. 2009), the mutation at the At2g18790 locus did not seem to directly disrupt or enhance photosynthetic capacity in our light response curve experiment. Taken together, we also learn that new phenotypes can be discovered thus demonstrating the benefits of full life cycle experiments integrating gene actions across the lifespan and across functional networks of genes. Identification of such outlier individual mutants serves as potential candidate genes for further inquiry and investigation in applied settings such as crop breeding.

### 4.4 Conclusions and Future Directions

This study quantitatively examined the effects of nutrient and temperature on single‐gene mutants and characterized rosette and reproductive traits in comparison to natural variation. Our findings suggest that mutants classified with seedling altered leaf size are influenced by both environmental conditions and genetic by environmental responses when examined at later stages of development. Increase and decrease sets of mutants maintained their rank order across manipulated environment treatments; however, increase lines were not consistently larger than the wildtype, emphasizing the importance of characterizing traits for all developmental stages. Quantitative measurements of leaf size mutants across manipulated environments highlighted tradeoffs between vegetative size and reproductive timing and output as increase mutants had similar rosette sizes to wildtype, but bolted faster, with generally greater fruit production and inflorescence height, which were enhanced in lower temperatures and nutrient addition.

Our experimental approach with ecological and genetic tools can have broader applications as context for environmental‐genetic studies of other annual plant species. As there are multiple complex environmental factors that influence plant traits in the wild, challenges from laboratory and field incongruencies are important constraints in understanding phenotypic variation (Arana and Picó 2025). Studying single‐gene mutants along with natural accession phytometers in controlled environmental treatments is an important initial step to bridge single‐gene effects and field populations. Here, we primarily employ natural accessions as phytometers for our experimental design, yet it also provides the opportunity for contextualizing our single‐mutant lines with natural variation that has been broadly reported in this species (e.g., Méndez‐Vigo et al. 2022). In some cases, single insert mutant variation is within, and sometimes more extreme than the natural accessions used here. Such context is helpful to connect responses of temperature and nutrients to natural environments of *A. thaliana* (e.g., Montesinos‐Navarro et al. 2010) and for other members of those plant communities. The response of conserved regulatory and genetic pathways to combined abiotic conditions found between Arabidopsis and other distant species creates opportunity to expand on environment‐genetic interactions outside of Arabidopsis systems (Arana and Picó 2025). Throughout the geographic range for many winter annual species, plants may grow larger earlier in the cool, wet portion of seasons before flowering when there is access to higher nutrients (Wolfe and Tonsor 2014). However, warmer temperatures during seasonal transitions may abruptly end the reproductive phase. Thus understanding the interaction effects between temperature and nutrient availability on plant performance for single genes can aid in extending these responses to other natural ruderal plant systems with small or large rosette size characteristics or predict when conditions become too warm for certain species to produce enough viable seed. Such patterns can negatively impact agricultural crop yield, for instance the oil seed crops which are closely related to Arabidopsis. In certain areas of agricultural production, nutrient runoff to surrounding areas may enhance floral production, but together with increasing temperatures may also facilitate floral abortion. Larger annual plants might generally produce more fruit, but interactions between temperature, nutrients and genetics constrain fruit development success in complex ways. Expanding to other ecologically important abiotic and biotic environmental treatments (e.g., Mukhaimar et al. 2024) would extend this work for other traits where among natural accession variation has been found. Additionally, new tests in mesocosms to validate the environmental studies presented here will further assist in application to broader plant species in agriculture and wild populations. With the trend of increases in annual global temperatures, our findings suggest that with a modest increase of only a few degrees C to a warmer average temperature, annual plants will experience overall less fruit production and a shift to greater floral abortion with 4°C increases in temperature, but that genotypic variation even at single gene level is also an essential consideration.

## Funding

This work was supported by the U.S. Department of Agriculture, 2019‐67033‐29241; National Science Foundation, 2121415, IOS‐1355106; College of Charleston.

## Conflicts of Interest

The authors declare no conflicts of interest.

## Supporting information


**Figure S1:** Box plots of increase, decrease, phytometer, and wildtype sets are plotted for standardized inflorescence height (*z* values) across nutrient and temperature treatments. Median is the horizontal line, the 25th–75th percentiles or interquartile range is the box, the interquartile range above the 75th or below the 25th percentile is the vertical line, outliers are points, blue rhombus are mean values. We detected differences between line sets (ndf = 2, ddf = 30.96, *F* = 6.418, *p* < 0.001), with the decrease set having shorter inflorescence height compared to increase set (df = 31, *t* = −3.303, *p* < 0.01). Nutrients and temperature significantly influenced inflorescence height (ndf = 1, ddf = 1621.97, *F* = 13.34, *p* < 0.001; ndf = 1, ddf = 1621.97, *F* = 28.11, *p* < 0.001 respectively). Temperature and line set factors had interaction effects on inflorescence height: taller inflorescence at higher temperature that is more pronounced in the increase set (ndf = 2, ddf = 1621.97, *F* = 4.996, *p* < 0.01).
**Figure S2:** Box plots of increase, decrease, phytometer, and wildtype sets are plotted for standardized fruit lengths (*z* values) across nutrient and temperature treatments. Median is the horizontal line, the 25th–75th percentiles or interquartile range is the box, the interquartile range above the 75th or below the 25th percentile is the vertical line, outliers are points, rhombus are mean values.
**Figure S3:** Scatter plot of standardized rosette diameter by standardized aborted fruits (*z* values) of increase, decrease, phytometer, and wildtype sets across temperature treatments. Square points and solid lines are the control nutrient treatment, and circle points and dashed lines are nutrient added treatments. We detected significant differences in slope for temperature and nutrient and line set (*p* < 0.0001).
**Figure S4:** Scatter plot of standardized rosette diameter by standardized fruit production (*z* values) of increase, decrease, phytometer, and wildtype sets across temperature treatments. Square points and solid lines are control nutrient treatments, and circle points and dashed lines are nutrient added treatments. There is a weak positive correlation between rosette diameter and fruit production across all line sets. The decrease set tends to have small rosettes and low fruit production. Across both controlled abiotic conditions, we detected significant differences in slope for rosette diameter and fruit production between increase and decrease sets (*p* < 0.0001).
**Figure S5:** Scatter plot of standardized rosette diameter by standardized inflorescence height (*z* values) of increase, decrease, phytometer, and wildtype sets *z* values across temperature treatments. Square points and solid lines are control nutrient treatments, and circle points and dashed lines are nutrient added treatments. There is a positive correlation between rosette diameter and inflorescence height, with larger rosettes, inflorescence height also becomes taller. The decrease set tends to have smaller rosettes and shorter inflorescences. We detected significant differences in slope for temperature and nutrient and line set (*p* < 0.0001).
**Figure S6:** Scatter plot of standardized rosette diameter by standardized days to bolting (*z* values) of increase, decrease, phytometer, and wildtype sets *z* values across temperature treatments. Square points and solid lines are control nutrient treatments, and circle points and dashed lines are nutrient added treatments.
**Figure S7:** Box plots of increase, decrease, phytometer, and wildtype sets are plotted for the standardized number of days to bolting (*z* values) across nutrient and temperature treatments. Median is the horizontal line, the 25th–75th percentiles or interquartile range is the box, the interquartile range above the 75th or below the 25th percentile is the vertical line, outliers are points, blue rhombus are mean values. There are significant differences across line sets (ndf = 2, ddf = 30.96, *F* = 4.629, *p* < 0.05), with the increase set shows overall faster reproductive timing than the decrease set (df = 31, *t* = 2.957, *p* < 0.05). Temperature and line set factors have some interaction effects: faster bolting at higher temperature for the increase set (ndf = 2, ddf = 1627.97, *F* = 10.020, *p* < 0.001).
**Figure S8:** Bar plots for rosette leaf area across temperature treatments for each mutant line. Error bars are one standard error. SALK_006410C (decrease line) and SALK_022035C (increase line) show an increase in the whole projected rosette area from 20°C to 24°C.
**Figure S9:** Mean rosette diameters of the mutant lines grown for the physiological measurements across the 4 treatments (20°C and 24°C, control and nutrient addition). Col‐0 (as COL70000) is the black circle line. The decrease lines are the solid lines. The increase set are the dashed lines. Using Col‐0 as a reference, each set has two mutant genotypes with different reaction norms; one accession showing clear decrease in rosette diameters from 20°C to 24°C, while the other accession showing clear increase in rosette diameters from 20°C to 24°C.
**Figure S10:** Leaf area adjusted gas exchange measurements graphed across each mutant line: SALK_006410C, SALK_022035C, SALK_022843C, SALK_096651C. Projected area used for adjustment is based on the separated rosette (leaves dissected and scanned individually). Each mutant replicate is paired with a wildtype (Col‐0) grown under the same environmental conditions. The dashed line represents plants grown in 24°C treatment and the solid line represents plants grown in 20°C treatment. Blue squares are lines in the decrease set, pink circles are lines in the increase set, and dark triangles are in the wildtype set (Col‐0). CO_2_ assimilation is ordered from increasing light intensities from 0, 250, 500, 750, 1000.
**Figure S11:** Mass adjusted gas exchange measurements graphed across mutant line: SALK_006410C, SALK_022035C, SALK_022843C, SALK_096651C. Each mutant replicate is paired with a wildtype (Col‐0) grown under the same environmental conditions. Projected mass used for adjustment is based on the entire rosette. The dashed line represents plants grown in 24°C treatment and the solid line represents plants grown in 20°C treatment. Blue squares are lines in the decrease set, pink circles are lines in the increase set, and dark triangles are in the wildtype set (Col‐0). CO_2_ assimilation is ordered from increasing light intensities from 0, 250, 500, 750, 1000.
**Figure S12:** Leaf area adjusted gas exchange measurements graphed across each temperature treatment for selected lines: SALK_006410C, SALK_022035C, SALK_022843C, SALK_096651C, Col‐0. Projected area used for adjustment is based on the entire rosette. Each mutant replicate is paired with a wildtype (Col‐0) grown under the same environmental conditions. The dashed line represents plants grown in 24°C treatment and the solid line represents plants grown in 20°C treatment. Blue squares are lines in the decrease set, pink circles are lines in the increase mutant set, and dark triangles are in the wildtype set (Col‐0). CO_2_ assimilation is ordered from increasing light intensities from 0, 250, 500, 750, 1000.
**Table S1:** Effects of line set (increase, decrease and wildtype/phytometer), temperature treatment, nutrient treatment and pairwise interactions for our inflorescence height and days to bolt. Individual genotypes (a.k.a. accessions) are nested within line set in this linear mixed model. Statistically significant factors are in gray cells.
**Table S2:** Gene information and Wilson‐Sánchez et al. (2014) leaf categories based on qualitative features compared to wildtype (Col‐0). The published qualitative relative seedling rosette size and leaf lamina rosette size were used to develop the line sets of increase and decrease used in our experiments. NR means that the trait was not reported in Wilson‐Sánchez et al. (2014) PhenoLeaf database.

## Data Availability

The phenotypic data used in this study are openly available on github in the form of an R package “unpakathon” https://github.com/stranda/unpakathon. These data can be accessed in R using: ‘devtools::install_github (“stranda/unpakathon”)’. Physiological data are available on FigShare https://doi.org/10.6084/m9.figshare.29647202.
