# Supplementary material for: Interactive Effects of Genes and Environment on Vegetative and Reproductive Tradeoffs of Leaf Size Mutants
Source: Plant Environ Interact. 2026 Aug 4;7(4):e70195. doi: 10.1002/pei3.70195 (PMC13438881; doi:10.1002/pei3.70195)
Supplement: Supplementary file 1 [file PEI3-7-e70195-s001.docx]

**Supporting Information**

**FIG. S1** Box plots of increase, decrease, phytometer, and wildtype sets are plotted for standardized inflorescence height (z values) across nutrient and temperature treatments. Median is the horizontal line, the 25th–75th percentiles or interquartile range is the box, the interquartile range above the 75th or below the 25th percentile is the vertical line, outliers are points, blue rhombus are mean values. We detected differences between line sets (ndf=2, ddf=30.96, F=6.418, p<0.001), with the decrease set having shorter inflorescence height compared to increase set (df=31, t=-3.303, p<0.01). Nutrients and temperature significantly influenced inflorescence height (ndf=1, ddf=1621.97, F=13.34, p<0.001; ndf=1, ddf=1621.97, F=28.11, p<0.001 respectively). Temperature and line set factors had interaction effects on inflorescence height: taller inflorescence at higher temperature that is more pronounced in the increase set (ndf=2, ddf=1621.97, F=4.996, p<0.01).

**FIG. S2** Box plots of increase, decrease, phytometer, and wildtype sets are plotted for standardized fruit lengths (z values) across nutrient and temperature treatments. Median is the horizontal line, the 25th–75th percentiles or interquartile range is the box, the interquartile range above the 75th or below the 25th percentile is the vertical line, outliers are points, rhombus are mean values.

**A)** Box plots for standardized fruit lengths along basal sections of main stem (z values) across nutrient and temperature treatments.

**B)** Box plots for standardized fruit lengths along mid sections of main stem (z values) across nutrient and temperature treatments.

**C)** Box plots for standardized fruit lengths along upper sections of main stem (z values) across nutrient and temperature treatments.

**FIG. S3** Scatter plot of standardized rosette diameter by standardized aborted fruits (z values) of increase, decrease, phytometer, and wildtype sets across temperature treatments. Square points and solid lines are the control nutrient treatment, and circle points and dashed lines are nutrient added treatments. We detected significant differences in slope for temperature and nutrient and line set (p<0.0001).

**FIG. S4** Scatter plot of standardized rosette diameter by standardized fruit production (z values) of increase, decrease, phytometer, and wildtype sets across temperature treatments. Square points and solid lines are control nutrient treatments, and circle points and dashed lines are nutrient added treatments. There is a weak positive correlation between rosette diameter and fruit production across all line sets. The decrease set tends to have small rosettes and low fruit production. Across both controlled abiotic conditions, we detected significant differences in slope for rosette diameter and fruit production between increase and decrease sets (p<0.0001).

**FIG. S5** Scatter plot of standardized rosette diameter by standardized inflorescence height (z values) of increase, decrease, phytometer, and wildtype sets z values across temperature treatments. Square points and solid lines are control nutrient treatments, and circle points and dashed lines are nutrient added treatments. There is a positive correlation between rosette diameter and inflorescence height, with larger rosettes, inflorescence height also becomes taller. The decrease set tends to have smaller rosettes and shorter inflorescences. We detected significant differences in slope for temperature and nutrient and line set (p<0.0001).

**FIG.** **S6** Scatter plot of standardized rosette diameter by standardized days to bolting (z values) of increase, decrease, phytometer, and wildtype sets z values across temperature treatments. Square points and solid lines are control nutrient treatments, and circle points and dashed lines are nutrient added treatments.

**FIG. S7** Box plots of increase, decrease, phytometer, and wildtype sets are plotted for the standardized number of days to bolting (z values) across nutrient and temperature treatments. Median is the horizontal line, the 25th–75th percentiles or interquartile range is the box, the interquartile range above the 75th or below the 25th percentile is the vertical line, outliers are points, blue rhombus are mean values. There are significant differences across line sets (ndf=2, ddf=30.96, F=4.629, p<0.05), with the increase set shows overall faster reproductive timing than the decrease set (df=31, t=2.957, p<0.05). Temperature and line set factors have some interaction effects: faster bolting at higher temperature for the increase set (ndf=2, ddf=1627.97, F=10.020, p<0.001).

**FIG. S8** Bar plots for rosette leaf area across temperature treatments for each mutant line. Error bars are one standard error. SALK_006410C (decrease line) and SALK_022035C (increase line) show an increase in the whole projected rosette area from 20C to 24C.

**FIG. S9** Mean rosette diameters of the mutant lines grown for the physiological measurements across the 4 treatments (20C and 24C, control and nutrient addition). Col-0 (as COL70000) is the black circle line. The decrease lines are the solid lines. The increase set are the dashed lines. Using Col-0 as a reference, each set has two mutant genotypes with different reaction norms; one accession showing clear decrease in rosette diameters from 20C to 24C, while the other accession showing clear increase in rosette diameters from 20C to 24C.

**FIG. S10** Leaf area adjusted gas exchange measurements graphed across each mutant line: SALK_006410C, SALK_022035C, SALK_022843C, SALK_096651C. Projected area used for adjustment is based on the separated rosette (leaves dissected and scanned individually). Each mutant replicate is paired with a wildtype (Col-0) grown under the same environmental conditions. The dashed line represents plants grown in 24C treatment and the solid line represents plants grown in 20C treatment. Blue squares are lines in the decrease set, pink circles are lines in the increase set, and dark triangles are in the wildtype set (Col-0). CO_2_ assimilation is ordered from increasing light intensities from 0, 250, 500, 750, 1000.

**FIG. S11** Mass adjusted gas exchange measurements graphed across mutant line: SALK_006410C, SALK_022035C, SALK_022843C, SALK_096651C. Each mutant replicate is paired with a wildtype (Col-0) grown under the same environmental conditions. Projected mass used for adjustment is based on the entire rosette. The dashed line represents plants grown in 24C treatment and the solid line represents plants grown in 20C treatment. Blue squares are lines in the decrease set, pink circles are lines in the increase set, and dark triangles are in the wildtype set (Col-0). CO_2_ assimilation is ordered from increasing light intensities from 0, 250, 500, 750, 1000.

**Fig. S12** Leaf area adjusted gas exchange measurements graphed across each temperature treatment for selected lines: SALK_006410C, SALK_022035C, SALK_022843C, SALK_096651C, Col-0. Projected area used for adjustment is based on the entire rosette. Each mutant replicate is paired with a wildtype (Col-0) grown under the same environmental conditions. The dashed line represents plants grown in 24C treatment and the solid line represents plants grown in 20C treatment. Blue squares are lines in the decrease set, pink circles are lines in the increase mutant set, and dark triangles are in the wildtype set (Col-0). CO_2_ assimilation is ordered from increasing light intensities from 0, 250, 500, 750, 1000.

**Table. S1** Effects of line set (increase, decrease and wildtype/phytometer), temperature treatment, nutrient treatment and pairwise interactions for our inflorescence height and days to bolt. Individual genotypes (a.k.a. accessions) are nested within line set in this linear mixed model. Statistically significant factors are in grey cells.

|  |  | **Inflorescence Height** | | | **Days to bolt** | | |
| --- | --- | --- | --- | --- | --- | --- | --- |
| **Mixed Model** | **Factor** | **Denominator df** | **F** | **P** | **Denominator df** | **F** | **P** |
| **Type 3 ANOVA Satterthwaite** |  |  |  |  |  |  |  |
|  | Line set | 30.96 | 6.4188 | **4.66E-03** | 30.96 | 4.6292 | **0.01742** |
|  | Temperature | 1621.97 | 28.1198 | **1.30E-07** | 1627.97 | 0.2233 | 0.63662 |
|  | Nutrient | 1621.97 | 13.3455 | **0.0002673** | 1627.96 | 4.0872 | **4.34E-02** |
|  | Temperature x Nutrient | 1621.97 | 1.6361 | 0.201041 | 1627.96 | 0.249 | 0.61786 |
|  | Line set x Temperature | 1621.97 | 4.9967 | **0.0068645** | 1627.97 | 10.0207 | **4.73E-05** |
|  | Line set x Nutrient | 1621.97 | 0.7477 | 0.4736287 | 1627.96 | 4.3864 | **0.01259** |
| **Random Effect** |  |  | **LRT** | **P** |  | **LRT** | **P** |
|  | Accession:line set |  | 1269.8 | **2.20E-16** |  | 1395.9 | **2.20E-16** |

**Table S2.** Gene information and Wilson-Sanchez et al. (2014) leaf categories based on qualitative features compared to wildtype (Col-0). The published qualitative relative seedling rosette size and leaf lamina rosette size were used to develop the line sets of increase and decrease used in our experiments. NR means that the trait was not reported in Wilson-Sanchez et al. (2014) PhenoLeaf database.

| **Accession** | **Gene Locus** | **Rosette Relative Size 18 day old seedlings from Wilson-Sanchez** | **Leaf Lamina Relative Size**  **18 day old seedlings from Wilson-Sanchez** | **Gene Description** |
| --- | --- | --- | --- | --- |
| SALK_006410C | At1g20580 | Decreased | Decreased | Small nuclear ribonucleoprotein family protein |
| SALK_016311C | At3g19360 | Decreased | Decreased | Zinc finger (CCCH-type) family protein |
| SALK_020615C | At1g76470 | Increased | NR | NAD(P)-binding Rossmann-fold superfamily protein |
| SALK_022035C | At2g18790 | Increased | NR | Red/far-red photoreceptor involved in the regulation of de-etiolation |
| SALK_022843C | At5g53500 | Decreased | Decreased | Transducin/WD40 repeat-like superfamily protein |
| SALK_026289C | At2g24090 | Decreased | Decreased | Ribosomal protein L35 |
| SALK_027847C | At3g12490 | Increased | Increased | Encodes a protein with cysteine proteinase inhibitor activity |
| SALK_037636C | At4g38600 | Increased | Increased | encodes a member of HECT ubiquitin protein ligase family that is involved in trichome cell morphogenesis |
| SALK_039252C | At1g33270 | Decreased | Decreased | Acyl transferase/acyl hydrolase/lysophospholipase superfamily protein |
| SALK_039359C | At2g04550 | Increased | Increased | Encodes a protein phosphatase that interacts with MPK12, but not with other MAP kinases |
| SALK_048175C | At1g30450 | Decreased | Decreased | member of Cation-chloride co-transporter family |
| SALK_057052C | At5g54770 | Decreased | Decreased | Encodes a thiamine biosynthetic gene that has a dual function in thiamine biosynthesis and mitochondrial DNA damage tolerance |
| SALK_057987C | At4g08900 | Increased | Increased | Encodes an arginase, likely to be involved in polyamine biosynthesis in pollen |
| SALK_059601C | At1g09520 | Increased | NR | NR |
| SALK_075661C | At3g13228 | Decreased | Decreased | RING/U-box superfamily protein |
| SALK_079466C | At1g02100 | Increased | Increased | Leucine carboxyl methyltransferase |
| SALK_094653C | At3g49040 | Increased | Increased | F-box/RNI-like superfamily protein |
| SALK_094856C | At1g63280 | Decreased | Decreased | Serine protease inhibitor (SERPIN) family protein |
| SALK_096651C | At5g11260 | Increased | Increased | Basic leucine zipper (bZIP) transcription factor |
| SALK_099684C | At5g06410 | Increased | NR | DNAJ heat shock N-terminal domain-containing protein |
| SALK_103278C | At4g32105 | Decreased | Decreased | Beta-1,3-N-Acetylglucosaminyltransferase family protein |
| SALK_104832C | At1g16470 | Increased | Increased | Encodes 20S proteasome subunit PAB1 (PAB1) |
| SALK_107544C | At4g23060 | Decreased | Decreased | IQ-domain 22 (IQD22); CONTAINS InterPro DOMAIN/s: IQ calmodulin-binding region (InterPro:IPR000048) |
| SALK_121366C | At3g25470 | Decreased | Decreased | bacterial hemolysin-related |
